# Suicidal Behavior in Adolescent Girls with Eating Disorders

**DOI:** 10.1192/j.eurpsy.2022.972

**Published:** 2022-09-01

**Authors:** N. Semenova, H. Slobodskaya, E. Rezun

**Affiliations:** 1 Scientific Research Institute of Medical Problems of the North, Department Of Child’s Physical And Mental Health, Krasnoyarsk, Russian Federation; 2 Federal State Budgetary Scientific Institution «Scientific Research Institute of Neurosciences and Medicine», Department Of Child Development And Individual Differences, Novosibirsk, Russian Federation

**Keywords:** Eating Disorders, girls, Suicide, Adolescents

## Abstract

**Introduction:**

Eating disorders are associated with suicidal behavior in adolescents. But the relationship between subthreshold eating disorders and suicidality is not well understood.

**Objectives:**

To examine suicidal behavior in adolescent girls with eating disorders (ED) and subthreshold eating disorders (SED).

**Methods:**

The study of 917 girls aged 12–17 used the Body Image and Eating Distress scale (Koskelainen et al., 2001) and questions about intentional self-harm (burns or cuts), suicidal thoughts and suicidal attempts. Adolescents were divided into three groups: girls with ED (n = 20); girls with SED (n = 88); and control group (CG, n = 809).

**Results:**

Self-harm was reported by 55% of girls with ED, 35.2% of girls with SED and 20.2% of CG girls (χ2 = 15.82, p <0.001). Suicidal ideation was reported by 65% of girls with ED, 51.1% of girls with SED and 27.2% of CG girls (χ2 = 21.86, p <0.001). Suicidal attempts were reported by 45% of girls with ED, 17.1% of girls with SED and 7.5% of the CG. There were no differences in the prevalence of self-harm and suicidal ideation between the ED and SED groups, rates of suicide attempts were 2.6 times higher in the ED group.

**Conclusions:**

Girls with both ED and SED have a high risk of suicidal behavior: 2.7-4.5 times higher rates of self-harm, 1.9-2.4 times higher rates of suicidal ideation, and 2.3-6 times higher rates of suicidal attempts. Management of such adolescents should include assessment of the risk of suicide.

**Disclosure:**

The study was supported by the Russian Science Foundation grant # 21-15-00033.

